# The Abolition of Secret Remedies

**Published:** 1905-12

**Authors:** 


THE ABOLITION OF SECRET REMEDIES.—When the American Medical Association undertook its proper duty of enlightening physicians as to the true composition of proprietary medicines, the step was hailed with delight by the profession, while, of course, there arose mutterings of anger from the camp of the unscrupulous manufacturers. Gradually the opposition is getting out into the light, so that it becomes possible to see who feels pinched by this great forward movement of the organized medical profession. Pharmaceutical manufacturers who have nothing to conceal welcome the advent of the Council on Pharmacy and Chemistry; but, unfortunately, in the last few years manufacturing and even retail pharmacy has become permeated with a spirit of deceit. For this state of affairs the ready gullibility of many thoughtless physicians is much to blame. To give the profession the actual facts is the task allotted to the Council on Pharmacy and Chemistry. Already physicians have been informed in regard to the acetanilid compounds, and seldom has a more important piece of work been laid before the profession. It is interesting to note that few, indeed, of the medical journals have mentioned the report of the Council on Pharmacy and Chemistry on the acetanilid compounds, though it appeared in the *Journal of the American Medical Association* over three months ago. The state of affairs is deplorable, but it is drawing to its close. Through it own State journals and through the *Journal of the A. M. A.*, the profession now has control of its means of publicity. The Council on Pharmacy and Chemistry will ascertain the truth about our drugs, and the profession's press will disseminate the knowledge. One of the greatest reform movements of our time it thus under way. Every physician will eagerly watch the march of coming events.—*Ohio State Medical Journal.* (In the journal combine; the Ohio "tentacle" of the Octopus.)

The *Journal of the A. M. A.* is, nevertheless, still carrying the advertisements of proprietary "secret" remedies, which ye editor confesses are *nostrums.* He says he will *drop them when the contracts expire!* Why did he ever make the contracts? Were these articles not as much "nostrums" then as now? If not, then when did they become "nostrums?" When did the spasm of virtue seize Mr. Simmons? It is a shame that the great (mis) representative organ of the medical profession of America should so stultify itself, and its editor be allowed to run with the hare—for revenue, —and hold with the hounds—for buncombe!

---

SENATORIAL AMENITIES: "The Ghost Bill." "Amend by substituting 'doctors' for 'paupers.' Let them dissect each other."—*Davidson* [would-be Lieutenant-Governor].

"If Jesus Christ were crucified today the doctors of Texas would buy or steal his body before night."—*Chambers.*

"The doctors of Fort Worth are the most ungodly set of butchers."—*Hanger.*

"The Osteopaths are right. You fellows are all wrong."—*Hicks,* in Twenty-ninth.

[In the Twenty-eighth Hicks and Davidson led the fight on the drugless fellows.]

